# Factors influencing the career choice and retention of community mental health workers in Ghana

**DOI:** 10.1186/s12960-015-0050-2

**Published:** 2015-07-09

**Authors:** Vincent I.O. Agyapong, Akwasi Osei, Conor K. Farren, Eilish McAuliffe

**Affiliations:** Department of Psychiatry, Faculty of Medicine and Dentistry, University of Alberta, Edmonton, Canada; Department of Behavioural Sciences, Kwame Nkrumah University of Science and Technology, Kumasi, Ghana; Centre for Global Health, University of Dublin, Trinity College, Dublin, Ireland; Ghana Mental Health Authority and Accra Psychiatric Hospital, Accra, Ghana; Department of Psychiatry, University of Dublin, Trinity College Dublin, Dublin, Ireland; School of Nursing, Midwifery and Health Systems, University College Dublin, Dublin, Ireland

## Abstract

**Background:**

Whilst there have been several studies exploring retention in health workers, little is known about health workers engaged in the provision of mental health services and the factors that affect their recruitment and retention.

**Aims:**

The objective of this research was to examine the views of stakeholders about the factors which influence career choices and retention of community mental health workers (CMHWs) in Ghana.

**Methods:**

We administered three separate, self-administered, semi-structured questionnaires to 11 psychiatrists, 29 health policy directors and 164 CMHWs across Ghana, including 71 (43.3%) community psychiatric nurses (CPNs), 19 (11.6%) clinical psychiatric officers (CPOs) and 74 (45.1%) community mental health officers (CMHOs).

**Results:**

Overall, 34 (20.7%) of all CMHWs chose to work in mental health because of the job prospects in mental healthcare. Overall, 12 (16.2%) CMHOs, 1 (5.3%) CPO and 20 (28.2%) CPNs reported they had considered leaving the mental health profession because of the stigma, with 4 (36.4%) psychiatrists and 12 (41.4%) health policy coordinators also reporting that they knew some CMHWs who had considered leaving the mental health profession because of stigma. Similarly, 16 (21.6%) CMHOs, 4 (22.1%) CPOs and 38 (53.5%) CPNs said they had considered leaving the mental health profession because of concerns about risk. Furthermore, 6 (54.5%) psychiatrists and 3 (10.3%) health policy directors said they knew some CMHWs who had considered leaving the mental health profession because of concerns about risk. Overall, 61 (37.2%) of CMHWs reported that they have considered leaving the mental health profession for other reasons other than stigma and risk including the following: the lack of support, respect and recognition from healthcare managers, lack of opportunities for professional development and poor conditions of service including low salaries, lack of office and personal accommodation and lack of risk allowance and transportation as well as poor inter-professional relationships.

**Conclusions:**

Several factors affect the recruitment and retention of CMHWs in Ghana, including the prospects of easy employment, stigma, risk, lack of opportunities for career progression and low salaries.

## Introduction

In order to tackle the workforce challenges specific to an organization, one must first understand the exact nature of those challenges. Across the entire economic spectrum, and especially in low- and middle-income countries (LAMICs) including Ghana, public sector resource allocation for mental health is disproportionately low [1], and this is from a health expenditure pie that is already much too small [2]. Close to a third of countries have no specified mental health budget, and of the 101 countries that have a mental health budget, most spend less than 1% of their total health budgets on mental healthcare [1]. Ghana’s mental health sector is funded primarily by the government and is supplemented by internally generated funds and donations. Total annual health expenditure is 7.8% of GDP (2011), per capita expenditure is US$ 114 and mental health funding receives just about 3% of the healthcare expenditure [3]. The low funding for mental health, among other factors, affects recruitment and retention of mental health professionals, and the scarcity of mental health professionals places specialist psychiatric care out of the reach of most people in LAMIC [4,5], particularly in the lowest income countries and in rural/low-income regions within countries [6]. Several other factors account for the relative lack of mental health professionals in LAMICs including insufficient training opportunities, deteriorating health of the workforce, brain drain and rural/urban imbalance [7]. The lack of education and training opportunities in mental healthcare severely handicaps LAMICs, as they have no means to make up for the scarcity of mental health professionals from an internal source [8]. The most common factors that force health workers away from jobs in rural areas are the lack of incentives and amenities, as well as limited opportunities for career progression [9]. The remote and underdeveloped areas with poor or no social amenities are always difficult to post staff to, without innovative incentives [10]. In an in-depth survey of nursing leaders and in-practice nurses in both rural and urban Ghana on potential incentives to promote recruitment and retention in rural service, many respondents reported low satisfaction with rural practice influenced by the high workload and difficult working conditions, perception of being “forgotten” in rural areas by the Ministry of Health, lack of professional advancement and the lack of formal learning or structured mentoring [11]. The issue of stigma and low prestige associated with mental health has also contributed to the relative lack of trained mental health professionals in LAMICs. For example, a study of Ghanaian medical students showed the students thought psychiatry had little prestige and was less lucrative than other specialties, and the majority felt uncomfortable interacting with patients with mental illness [12].

In another study in Ghana to assess how mental health workers perceive their jobs and what drives them to work in mental healthcare, respondents in both clinical care and administrative support roles expressed a desire to leave the field of mental health in order to further their careers. They reported that working in a psychiatric hospital was seen as a stepping stone to careers outside of public mental healthcare, such as a job in private medical care, human resource management or business [13].

Brain-drain issues also contribute greatly to the mental health capacity problem in LAMICs. Migration of professionals from low- and middle-income countries to richer countries is a large-scale phenomenon [14-17], and in 2000, it was estimated that there were 1.5 million professionals from LAMICs working in industrialized countries [18]. Like all other health professionals in these countries, mental health providers often migrate to areas with higher incomes [8].

To address the human resource gap within Ghana’s mental health delivery system, in November 2007, Ghana’s Ministry of Health decided to create two new community-based mental health posts and develop curricula to support these. The two posts were the clinical psychiatry officer (CPO) and the community mental health officer (CMHO). CPOs are trained for 2 years and are expected to diagnose and treat a limited number of mental health conditions whilst CMHOs are trained for 1 year and are only expected to be involved in case detection for referral to CPOs and psychiatrists, health promotion and monitoring with treatments initiated by CPOs and psychiatrists [19]. Currently, due to the limited numbers of psychiatrists who practice mainly in three big cities in Ghana, community mental healthcare is provided predominantly by community mental health workers (CMHWs) comprising CPOs, CMHOs and community psychiatric nurses (CPNs). Whilst there have been several studies exploring retention in health workers, little is known about health workers engaged in the provision of mental health services and the factors that affect their career choices and retention [20]. Thus, we seek to examine the views of the CMHWs, psychiatrists and health policy directors around these issues and to make recommendations to improve the recruitment and retention of these mental health cadres. The two research questions were the following:

1. What factors influence CMHWs to choose careers in mental health?
2. What factors influence the retention of CMHWs in Ghana?

**Figure 1:**
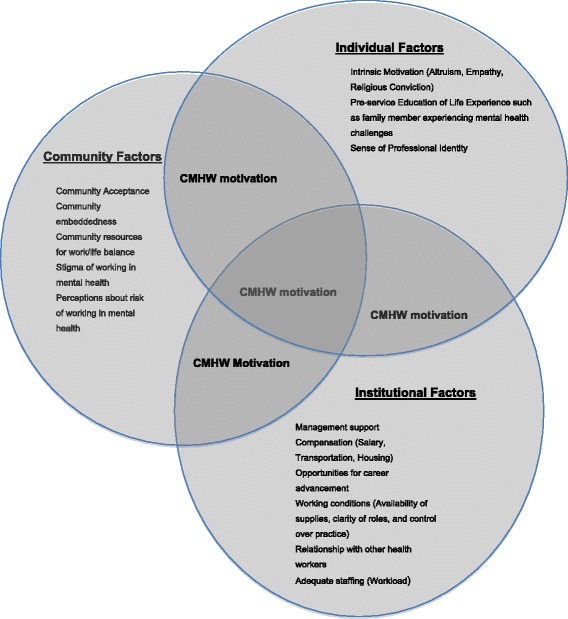
Conceptual framework of factors influencing CMHW career choice and retention in a task-shifting model of care.

The conceptual framework was based on empirical evidence suggesting that community health worker motivation to work in mental health is based on an interaction between community, institutional and individual factors [11,13,21] as shown in Figure 1.

## Methods

### Study setting

The study was conducted in Ghana, a small tropical country in West Africa with a total land area of about 239 000 square km and a population of about 25 million people. As of 2011, there were three mental health hospitals in the country with a total of 1322 beds (5.5 beds per 100 000 population), two of which are located in Accra, the capital city, and the other located in the Central Region of Ghana which also is in the south of the country. All of the hospitals are organizationally integrated with mental health outpatient facilities. The total number of human resources working in mental health facilities per 100 000 population is 7.82. The breakdown according to profession is as shown in Table 1 [3].

**Table 1:** **Total number of human resources working in mental health in Ghana in 2011**

**Mental health professional**	**Total number**	**Number per 100 000**
Psychiatrists	18	0.07
Other doctors not specializing in psychiatry	31	0.13
Nurses	1256	5.19
Clinical psychologists	19	0.08
Occupational therapists	4	0.02
Social workers	21	0.09
Other mental health workers	546	2.25

Of the 18 psychiatrists, 12 were in active public service with the rest on retirement. The “other mental health workers” in Table 1 included auxiliary staff, health assistants, medical assistants, community mental health officers, community psychiatric officers and professional and paraprofessional psychosocial counsellors [3].

### Study design

The study design was a cross-sectional survey with mixed quantitative and qualitative methods with both the quantitative and qualitative data being collected in paper and pen format. The qualitative data comprised responses to open-ended questions on the survey form. Purposive sampling was used to select CMHW respondents from all the 10 regions of Ghana so as to ensure regional balance in the responses whilst a convenience sampling approach was used for data collection from CMHWs within each region and for the health policy directors due to the geographical spread of these respondents. Total population sampling approach was used for data collected from the psychiatrists due to their relatively small numbers.

### Ethics and institutional approval

The study received prior institutional review board approvals from the Health Policy and Management and Global Health Ethics Committee at Trinity College Dublin (Reference number: 07/2013/06) and the School of Medical Sciences, Kwame Nkrumah University of Science and Technology, Kumasi, Ghana (Reference number: CHRPE/AP/300/13). The study also received the approval of the office of the Chief Psychiatrists of the Ghana Health Service. All study participants received information leaflets about the study before completing the questionnaires. Written informed consent was obtained from all study participants before they completed the survey questionnaires.

### Data collection

Initially, three separate but similar self-administered, semi-structured questionnaires with optional answers, including Likert scales and open-ended questions, were developed by the research team based on a review of literature on similar studies assessing the extent and impact of task shifting as well as the challenges, including factors, which influence the recruitment and retention of community health workers. No validated instruments to measure these concepts in this population could be found in the literature. All the three questionnaires included questions exploring issues including the following: Why do CMHWs in Ghana choose to work in mental health? How do stigma, risk and conditions of service (pay and remuneration) affect the retention of CMHWs? What support do CMHWs get from district health management teams (DHMTs), and how does this influence their retention? Which other factors affect the retention of CMHWs in Ghana? The questionnaires were pretested on two respondents in each category. They were revised to include more Likert scales based on the results of the pretest before being administered to the respondents. They generally took 15 min to complete, and no monetary or other incentives were provided to the respondents.

**Figure 2:**
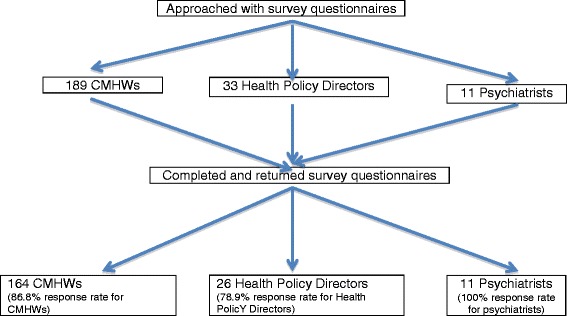
Flow chart for study participants.

Data were collected between the 10th of August 2013 and 30th of October 2013. Data from CMHWs nationwide were obtained with the assistance of the national coordinator of the CPNs and her 10 regional representatives as well as a CPO who works as a lecturer at the College of Health and Wellbeing at Kintampo. The former distributed and returned all 109 questionnaires to CMHWs working in all 10 regions through her regional coordinators. The latter administered the questionnaires to 80 CMHWs attending a conference at the College in August 2013 of which 55 questionnaires were returned. The overall response rate from CMHWs was therefore 86.8%. Data from all the psychiatrists and health policy directors were collected by the lead investigator (VIOA) through face-to-face contact. In the case of the health policy directors, comprising mainly of district and regional directors of health services, the lead investigator approached 33 of them at a regional performance review meeting organized by the Eastern Regional Health Directorate and two other health coordinators working in another region of which 26 agreed and completed the survey questions giving a response rate for health policy directors of 74.28%. All the psychiatrists who were approached consented and participated in the study, a response rate of 100% (Figure 2).

### Data analysis

Quantitative data were analysed using descriptive and inferential statistics with SPSS version 20. We compared some of the responses offered by the different CMHWs using chi-square and Fisher’s exact tests. Qualitative data were compiled from the responses to open-ended questions on the survey forms and then subjected to a thematic analysis, with coding being performed manually.

## Results

### Reasons for choosing a career in mental health

**Figure 3:**
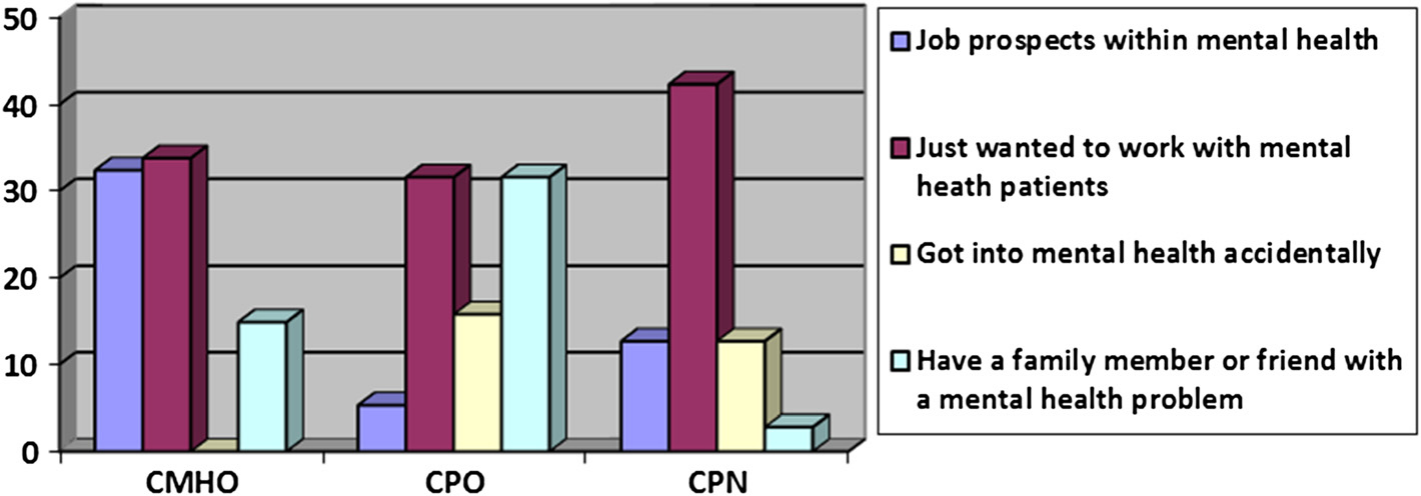
Percentages of the CMHWs groups and the reasons they chose to work in mental health (df = 8, *P* value =0.00).

Respondents were given four choice options: 1) job prospects within mental health, 2) I just wanted to work with mental health patients, 3) I got into mental health accidentally and 4)I have a family member or friend with a mental problem and was touched by their plight and an “other” category where they could specify additional reasons. A comparison of the reasons why the CMHWs chose to work in mental health was made using cross tabulation. The results of this analysis showed there was as a statistically significant association between the CMHW type and the reasons they chose to work in mental health as shown in Figure 3.

Figure 3 suggests that significantly more CMHOs choose to work in mental health because of the job prospects (32.4%) compared to both CPOs (5.3%) and CPNs (12.7%). Furthermore, no CMHO got into the mental health profession accidentally. Around a third of all CMHWs said they just wanted to work in mental health with fairly similar proportions from the different CMHW types in this group. However, a higher proportion of CPOs (31.6%) chose to work in mental health because they had a family member or friend with mental health problems compared with CMHOs (14.9%) or CPNs (2.8%).

Other reasons given by the CMHWs for choosing a career in mental health can be grouped under three themes, namely: touched by the plight of mental health patients, developed an interest in mental health after undertaking an attachment in a psychiatric hospital and desire to learn more about mental health. Some of the reasons under the various themes are as summarized in Table 2.

**Table 2:** **Other reasons why CMHWs chose careers in mental health**

**Theme**	**Reasons**	**Source**
Touched by the plight of mental health patients	“I was touched by the plight of a mental health patient who lived near my house. I felt they deserved better”	CMHO
	“The desire to care for the mentally ill so that they can recover and go back into society and be able function normally and feel a part of society”	CPO
	“I just wanted to help the unfortunate patient who no one wanted to be associated with”	CPN
	“I chose mental health because everybody seemed to go the other way round making the mental sector a neglected area”	CPN
	“I wanted to care for the sick and I thought society had failed the mentally ill so I thought I could make a difference there”	CMHO
	“I just wanted to help reduce mental health problems”	CMHO
Developed an interest in mental health after undertaking an attachment in a psychiatric hospital	“I was introduced to mental health during my affiliation and developed interest”	CPN
	“It became an area of interest after an attachment to the Ankaful Psychiatric hospital as a Community Health Nurse”	CPO
	“I was touched by the plight of mental patients during nursing affiliation”	CPN
Desire to learn more about mental health	“I was interested in knowing the cause of mental illness and why people get the mental conditions”	CMHO
	“I wanted to learn the art of caring for mentally ill patients and to help others understand mental conditions”	CPO
	“I was interested in learning about behaviours of individuals, especially abnormal behaviours”	CPN

### Concerns about the state of mental healthcare in Ghana

**Figure 4:**
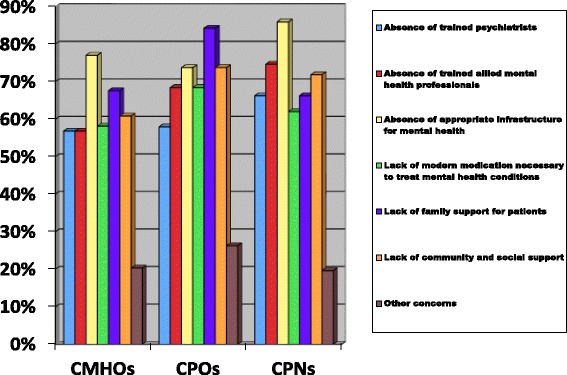
Percentage of the different CMHWs who expressed specific concerns about the state of mental healthcare in Ghana.

Overall, 89.2% and 97.3%, respectively, of CMHOs expressed concern about the state of mental healthcare in Ghana before and after they began to work in mental health. For CPOs, it was 89.5% and 100%, respectively, whilst 88.7% and 97.2%, respectively, for CPNs expressed concerns about the state of mental healthcare before and after they began to work in mental health. Figure 4 shows the specific concerns expressed by the different CMHWs.

Respondents were given a number of optional responses and asked to tick all those that applied. They were also given the opportunity to add additional concerns. The majority of CMHWs expressed concern about the lack of an appropriate mental health infrastructure followed by the lack of family support for patients and then the lack of community and social support. The other concerns identified by the community mental health workers included the following: stigmatization of mentally ill clients, lack of motivation and support for mental health professionals, cost of some psychotropic drugs and lack of knowledge about appropriate place to get mental health services.

### Impact of stigma, risk and other factors on the mental health workforce

**Figure 5:**
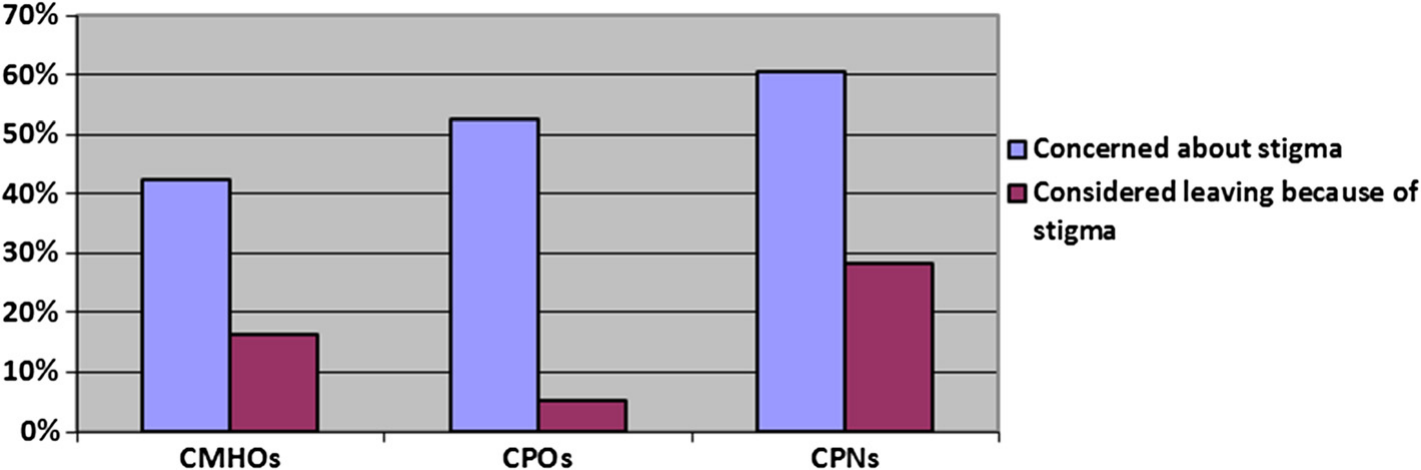
Percentages of CMHWs who expressed concerns about the risk associated with working in mental health and those who had considered leaving the mental healthcare profession because of concerns about stigma.

A five-point Likert scale was used to assess respondents’ perception as to whether there is stigma associated with working in mental health, the extent to which they are affected by this stigma and the extent to which it might have caused them to consider leaving their job. Almost all the CMHWs (163 (99.4%)) reported that they believed there is stigma associated with working in mental health. The CMHWs were also asked if they had been impacted negatively by the stigma associated with working in mental health and if they had considered leaving the mental health profession because of this stigma. The results are as summarized in Figure 5.

However, only 35 (42.5%) of CMHOs, 10 (52.6%) of CPOs and 53 (60.6%) of CPNs reported they have been negatively impacted by the stigma in mental health. Furthermore, only 12 (16.2%) of CMHOs, 1 (5.3%) CPO and 20 (28.2%) of CPNs reported they had considered leaving the mental health profession because of the stigma. On the other hand, all the psychiatrists believed that all CMHWs are affected by stigma although only four (36.4%) of the psychiatrists said they knew some CMHWs who had considered leaving the mental health profession because of stigma. Again, 16 (55.2%) health policy coordinators were of the opinion that CMHWs are affected by stigma with 12 (41.4%) of them reporting that they knew of some CMHWs who had considered leaving the mental health profession because of stigma.

**Figure 6:**
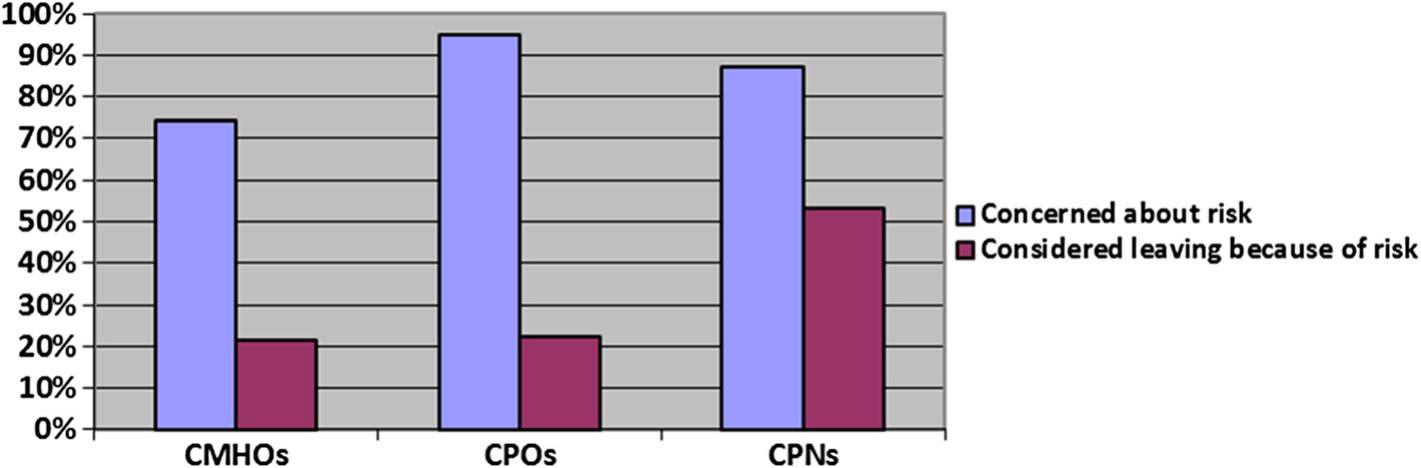
Percentages of CMHWs who expressed concerns about the risk associated with working in mental health and those who had considered leaving the mental healthcare profession because of concerns about risk.

The CMHWs were also asked if they had concerns about the risk associated with working in mental health and if they had considered leaving the mental health profession because of this risk. The results are as summarized in Figure 6.

On the other hand, all the 11 psychiatrists believed that CMHWs are affected by the risk of working in mental health, although only 6 (54.5%) of the psychiatrists said they knew of CMHWs who had considered leaving the profession because of concerns about risk. Furthermore, 26 (89.7%) health policy coordinators also believed that CMHWs are affected by risk but only 3 (10.3%) said they knew of some CMHWs who had considered leaving the mental profession because of concerns about risk.

Overall, 61 (37.2%) of the CMHWs reported that they have considered leaving the mental health profession for other reasons other than stigma and risk including the following: the lack of support, respect and recognition from healthcare managers, lack of opportunities for professional development and poor conditions of service including low salaries, lack of office and personal accommodation and lack of risk allowance and transportation as well as poor relationships with other healthcare workers including psychiatrists and district medical officers. One CMHO wrote, “I have worked for two years and have still not received any salary”. One CPO also wrote, “There is no motivation and I have had to use my own means of transport and resources to deliver services without any vehicle maintenance allowance being paid to me”. Another CPO wrote, “there is conflict between me and the medical officer in my hospital”. Similarly, one of the CPNs wrote, “there is poor relationship between the psychiatrist and community psychiatric nurses”. Another CPN also wrote, “The mental health profession is neglected by the health authorities and is not given the necessary logistics and assistance to aid its workforce”. Similarly, seven (63.6%) of the psychiatrists said they also knew of some community mental health workers who had considered leaving the mental health profession for other reasons including low salaries, lack of adequate protection against risk and lack of opportunities for career progression, in-service training and further education. Furthermore, 11 (37.9%) health policy coordinators said they knew of some CMHWs who had considered leaving the mental health profession for other reasons including the lack of motivation, low salaries and after getting opportunities to enrol in other health training institutions.

### Support from district health management teams and conditions of services

The CMHWs were asked to indicate on a five-point Likert scale the extent to which they felt supported by the DHMTs in which they work. Overall, only 35.6% of CMHOs, 47.4% of CPOs and 45.1% of CPNs reported they feel supported by their DHMTs. A chi-square test for independence indicated no significant association between the CMHW types and the response regarding support from the DHMT (*P* = 0.41). In contrast, five (45.5%) psychiatrists said the DHMTs support CMHWs to some extent, four (36.4%) said they support them only to a limited extent whilst two (18.2%) said the DHMT do not support the CMHWs at all. However, all the health policy coordinators and implementers believed that the CMHWs feel supported by the DHMTs although to varying extents, including 12 (41.4%) who said they support them to a limited extent, 9 (31%) who said they support them to some extent and 8 (27.6%) who said they absolutely support them.

Only 5 (6.8%) of CMHOs, 7 (36.8%) of CPOs and 15 (21.1%) of CPNs thought they receive adequate remuneration for the work they do. Consistent with this, 33 (43.2%) CMHOs, 7 (36.8%) CPOs and 37 (52.1%) CPNs said they will probably leave the mental health profession if they find a better paying job in other sectors of the economy. Overall, 162 (98.8%) of the mental health workers reported that they believed the district health management teams could do more to support their work. The CMHWs identified several incentives and support that they could get from the DHMT to enhance their work including the provision of transportation and other logistics such as umbrellas, rain coats and public address systems for community outreach work, office and personal accommodation, regular supply of medication, security for staff visiting patients in their homes and payment of risk allowance; giving similar recognition to mental health as is given to physical health and integration of mental health into public health activities; and regular in-service training and supervision as well as better infrastructure for mental healthcare. Overall, seven (63.6%) of the psychiatristscompared to five (17.2%) of the health policy coordinators reported that they do not think CMHWs receive adequate pay and incentives for the work they do in their districts. Also, all the 11(100%) psychiatrists compared to 24 (82.8%) of the health policy coordinators were of the opinion that if the CMHWs got better paid jobs in other sectors of the economy, they will leave their current jobs. Incentive packages identified by the psychiatrists which could enhance the work of the CMHWs included the following: transportation, enhanced pay, office and residential accommodation, risk allowance, early retirement, improved work environment, recognition for human resource development, monetary incentives, early promotion, free utility services and accommodation and they should be treated like all other healthcare professionals. Similarly, health policy coordinators identified incentive packages which they think could enhance the work of the CMHWs including the following: means of transportation, opportunities for career progression and further training, enhanced supervision, enhanced salaries, office and residential accommodation, free utility services, risk allowance and early promotion. One district director of health wrote, “Salary should be one step ahead of those in equivalent ranks in other health professions”.

## Discussion

Attrition rates for community health workers (CHWs) of 3.2% to 77% are reported in the literature [22]. One review [23] found attrition rates of 30% over 9 months in Senegal and 50% over 2 years in Nigeria. Whilst the attrition rates among CMHWs in Ghana is not known, it can be assumed from the above international data to be significant. It is therefore essential that efforts are made by the government of Ghana to recruit well-motivated, committed and well-resourced CMHWs to cater for the mental health needs of their communities.

### Motivation for joining the mental health workforce

There are several factors which motivate people to join the mental health workforce. Our results suggest that there are statistically significant differences in the reasons which motivated the three groups of health cadres in Ghana to join the mental health workforce, with significantly more CMHOs than CPOs and CPNs joining the mental health workforce because of the prospects of gaining employment. CMHOs are the least trained of all the CMHWs and the high youth unemployment rate in Ghana may be what drives some people to join this cohort of health professionals. Some CMHWs chose to work in mental health because they simply wanted to work with mental health patients or cared about the plight of mental health patients. This is exemplified by the remarks of one of the CMHOs who wrote (Table 2), “I was touched by the plight of a mental health patient who lived near my house. I felt they deserved better”. Reasons for choosing to work in mental health are important when considering retention of the health cadres as those who joined the mental health workforce because they wanted to work with mental health patients are likely to stay even if they are offered more lucrative opportunities in other sectors compared to those who joined because it simply presents an opportunity for employment. Furthermore, candidates who are unprepared for the job’s day-to-day challenges are more likely to leave the position at the least opportunity and so potential applicants should not be kept in the dark about the real nature of the work so that they can make informed choices [21]. It may therefore be necessary for health policy coordinators and heads of community mental health training institutions to interview applicants as part of the admission process so that their motivation for seeking to join the mental health workforce can be established. In addition, skills and qualities that health policy coordinators are looking for in workers could be translated into interview questions. For example, they may want to ask why a candidate is interested in the mental health field and probe for past experiences with friends or family members who have experienced mental illness [21]. This can potentially help stem the attrition within the mental health workforce.

### Impact of the state of mental healthcare, stigma, risk on recruitment and retention of CMHWs

Our results suggest that about 90% of all CMHWs in Ghana expressed concerns about the poor state of mental healthcare in Ghana before they even joined the mental healthcare profession. This suggests that the poor state of mental healthcare in itself did not deter these categories of health cadres from choosing to work in this field. The majority of CMHWs expressed concern about the state of the mental health infrastructure which is a reflection of the very low and inadequate budgetary support for mental health from the central government. Furthermore, the majority also expressed concern about the lack of family, community and societal support for those with mental health difficulties. This, however, seems to be the driving force for why some of the CMHWs chose to work in mental health as exemplified by the CPN who wrote, “I just wanted to help the unfortunate patients who no one wants to be associated with”.

Many studies have been conducted on the effects of stigma and discrimination against persons with mental health difficulties and their families [24-29]. However, not as many studies have examined stigma against mental health workers. The vast majority of stakeholders including CMHWs, psychiatrists and health policy coordinators believed there is stigma associated with working in mental health; however, only about half of the CMHWs said they have been impacted by the stigma in mental health. Even more worrying is the fact that about one in every five CMHWs reported that they have considered leaving the mental health profession because of stigma. In addition, one in three psychiatrists and two in every five policy coordinators reported they knew of CMHWs who had considered leaving the mental health profession because of stigma. Stigma is therefore an important factor in the recruitment and retention of CMHWs. In a study in Belgium to examine the degree of stigmatization among trainee psychiatrists, 75% of all trainee psychiatrists confirmed hearing denigrating or humiliating remarks about the psychiatric profession more than once [30]. Stigma also prevents Ghanaian medical students from choosing psychiatry as a career option [12] and therefore needs a significant response at the policy level if the critical shortage of mental health workers in Ghana is to be comprehensively addressed. Stigma could be tackled through an improvement in the infrastructure for mental healthcare and a comprehensive programme of public education.

As with stigma, the majority of stakeholders believed there is risk associated with working in mental health with one in every three CMHWs reporting they had considered leaving the mental health profession because of concerns about risk. Furthermore, 1 in every 2 psychiatrists and 1 in every 10 health policy coordinators reported they knew of a CMHW who had considered leaving the mental health profession because of concerns about risk. This makes concerns about risk an important consideration in the recruitment and retention of CMHWs. These concerns also need a policy response as it has resource implications. Security arrangements need to be adequate for all settings in which the CMHWs provide their services. In addition, the payment of risk allowance or enrolment in some form of employment-related insurance will go a long way to alleviate these concerns.

### Impact of conditions of service on recruitment and retention of CMHWs

Whilst CHWs’ success rate is often lauded in the early stages of a new and exciting project, their motivation diminishes over time unless frequent steps are taken to maintain their enthusiasm for their essential services [31]. Apart from concerns about stigma and risk, many stakeholders, including a third of all the CMHWs, identified other reasons why they had considered leaving the mental health profession, including the following: the lack of support, respect and recognition from healthcare managers, lack of opportunities for professional development and poor conditions of service including low salaries, lack of office and personal accommodation and lack of risk allowance and transportation as well as poor relationships with other healthcare workers including psychiatrists and district medical officers. Our results are consistent with previous surveys that uncovered that lack of advancement, work overload, poor salary, too few staff and poor organizational culture are the top challenges for healthcare workers [11,32,33]. A frequent cause of employee dissatisfaction and turnover is the lack of a well-formed career ladder for mental health workers [21,32].

In a task-shifting model, additional incentives are perceived as capable of reinforcing the potential motivation and ultimately increase health workers productivity [34]. Advanced degrees are another means of creating advancement potential. Rewarding employees for obtaining such degrees, for example, by increasing salary, benefits and status within the organization, encourages employees to stay with the job longer and increase their knowledge.

The majority of CMHWs and psychiatrists but not health policy coordinators were of the opinion that CMHWs in Ghana do not receive adequate remuneration for the role they play within Ghana’s mental health delivery system. Furthermore, all the psychiatrists and the majority of the health policy coordinators were of the opinion that if the CMHWs got better paid jobs in other sectors of the economy, they will leave their current jobs. A previous study in Ghana suggests that health workers in the private sector receive better pay and incentives than their counterparts working in the public sector [35]. This should be a source of worry to the government and health policy planners. It is a call to action which requires a policy response to ensure that significant incentives such as those identified by the stakeholders are offered to those who choose to work in mental health. Ghana’s Ministry of Health has an existing deprived area incentive scheme to attract, retain and sustain staff in rural areas including discriminatory fellowship allocations in favour of those working in deprived areas and a reduced period for promotion by 1 year [36]. However, these incentives are targeted at mid-level health workers rather than lower health cadres including CMHWs. Zambia’s “Health Workers Rural Retention Scheme”, which includes housing or housing allowances, fast track promotion and career development opportunities, car or car loans and education grants for staff children among other things, is seemingly successful in keeping health workers including community health workers in underserved areas [10]. It will therefore be useful for Ghana’s Ministry of Health and the Ghana Mental Health Authority to expand on the existing deprived area incentive scheme to include CMHWs. Finally, there is a need for regular staff surveys and periodic monitoring and review of the attrition data within each district to identify ongoing factors which influence the attrition among CMHWs.

### Limitations

A limitation of our study is that although the survey questionnaire was well researched based on empirical literature from previous studies in the field and pretested before use, it is nonetheless not a validated instrument. Furthermore, the paper and pen approach adopted in the survey might have limited the responses of the respondents to some of the open-ended questions, the effect of which has been that the results are predominantly quantitative in nature with limited qualitative data. Another limitation was that some of the potential respondents did not consent to participate in the study which raises the possibility of response bias among the respondents. Finally, although it would be interesting to examine the strength of the association between independent variables and dependent variables such as considerations about CMHWs joining or leaving the mental health profession, our study was not designed to explore these issues. The study was designed to be exploratory in nature and used a predominantly quantitative tool to allow ease of data collection across the different cadres offering mental health services. These limitations notwithstanding, a nationally representative sample of CMHWs and psychiatrists were included in the study which is a strength of this study.

## Conclusions

Our study has identified several factors that affect career choice and retention of CMHWs in Ghana, including the prospects of easy employment, stigma, risk, lack of opportunities for career progression and low salaries. Mental health policy makers and heads of mental health training institutions need to screen prospective students to ascertain their motivation for wanting to train as CMHWs before they are enrolled in the programmes. Ghana’s Ministry of Health and the Ghana Mental Health Authority also need to comprehensively address the factors that have the potential to decrease attrition rates within these health cadres by expanding on the ministry’s existing deprived area incentive scheme to provide enhanced security and logistics as well as enhanced salaries and clear career paths for CMHWs.

## Abbreviations

LAMICs: Low- and middle-income countries
DHMT: District health management team
CMHW: Community mental health worker
CMHO: Community mental health officer
CPO: Clinical psychiatric officer
CPN: Community psychiatric nurse
